# Study of the Distortion of the Indirect Angular Measurements of the Calcaneus Due to Perspective: In Vitro Testing

**DOI:** 10.3390/s21082585

**Published:** 2021-04-07

**Authors:** Isidoro Espinosa-Moyano, María Reina-Bueno, Inmaculada C. Palomo-Toucedo, José Rafael González-López, José Manuel Castillo-López, Gabriel Domínguez-Maldonado

**Affiliations:** 1 Department of Podiatry, Faculty of Nursing, Physiotherapy and Podiatry, Universidad de Sevilla, C/Avice-na s/n, 41009 Seville, Spain; espinosa@us.es (I.E.-M.); ipalomo@us.es (I.C.P.-T.); jmcastillo@us.es (J.M.C.-L.); gdominguez@us.es (G.D.-M.); 2 Department of Nursing, Faculty of Nursing, Physiotherapy and Podiatry, Universidad de Sevilla, C/Avenzoar nº6, 41009 Seville, Spain; joserafael@us.es

**Keywords:** biomechanics, instrumentation, image processing, computer-assisted, foot, gait

## Abstract

The study of the foot is relevant in kinematic analyses of gait. Images captured through a lens can be subjected to various aberrations or distortions that affect the measurements. An in vitro study was performed with a rearfoot simulator to compare the apparent degrees (photographed) with the real ones (placed in the simulator) in the plane of the rearfoot’s orientation, according to variations in the capture angle in other planes of space (the sagittal plane and transverse plane—the latter determined by the foot progression angle). The following regression formula was calculated to correct the distortion of the image: real frontal plane = 0.045 + (1.014 × apparent frontal plane) − (0.018 × sagittal plane × foot progression angle). Considering the results of this study, and already knowing its angle in the transverse and sagittal planes, it is possible to determine the angle of a simulated calcaneus with respect to the ground in the frontal plane, in spite of distortions caused by perspective and the lack of perpendicularity, by applying the above regression formula. The results show that the angular measurements of a body segment made on frames can produce erroneous data due to the variation in the perspective from which the image is taken. This distortion must be considered when determining the real values of the measurements.

## 1. Introduction

The quantification of the biomechanical characteristics of a person’s gait is an important clinical tool for evaluating normal and pathological patterns of locomotion [1].

There are several methods to assess the gait pattern, classified into two broad categories, namely: kinetics and kinematics. The kinetic analysis is used to determine the joint moments and forces involved in walking, such as the ground reaction force. The kinematic analysis depicts the movement patterns without considering the forces involved in motion production, but instead considering the spatiotemporal variables [2].

Basically, there two types of kinematic analysis: 2 dimensional (2D) and 3 dimensional (3D). In 3D gait analysis, the basic idea is to record the movement, using markers attached to the segment studied, from different positions and, by triangulation, represent it graphically in the three planes of space. This requires an integrated system of markers, cameras, computer equipment, and programs. Often used for scientific research, the use of the 3D method is limited in clinical practice because of aspects such as its high cost, long evaluation, and/or processing time [3,4].

In 2D gait analysis, a photo or video camera is used, and the movement is analyzed in a single plane of space. Two-dimensional video analysis is commonly used to assess kinematics when three-dimensional motion capture is unavailable. Its applicability is for clinical and scientific research. Its cost is much lower than for 3D gait analysis [5].

The study of the foot is relevant in the kinematic analysis of the gait, but if it is not done through the appropriate technological methodology, it is difficult to obtain conclusive results. Solutions to this problem have been available for some time: to capture images of the movement to be studied and analyze them in detail, manipulating the viewing speed and using the time and the auxiliary means necessary to study each gait gesture [6].

A high-speed camera can be used to evaluate the foot strike pattern [7,8]. The time can be obtained and quantified via the identification of the key moments in gestures and freeze framing. The time can be calculated via the number of frames between moments, with knowledge of the number of frames per second of the recording, and the angles can be measured using a computer program (supported by references previously marked on the foot). The identification of key moments in high-frequency recordings is possible because of the greater number of images per second, which means these moments can be determined accurately enough. Likewise, the measurement of the angles is facilitated as, given the high number of images, these are stable, enabling the viewing of the references with sufficient clarity [9].

However, in the information provided later, it is noted that errors of perspective are one of the main problems. This has special significance in the study of the position of the calcaneus with respect to the ground. This anatomical area mainly exhibits interpersonal deviations in the transverse plane, caused by differences in the gait angle of each subject —foot progression angle (FPA; the long axis of the foot and the walking direction) [10,11,12,13]. This happens because of the impossibility of framing the camara in line with the axis of the foot without interposing the other foot during the gait.

The image captured through a lens can be submitted to a series of aberrations or distortions that influence the measurements. The distortions of perspective affect the image’s trustworthiness. Conic perspective is applied in the photographic technique. As in the human retina, when viewed via a conic perspective, more distant objects appear smaller than closer ones. This hinders the quantitative analysis of the images [14]. Regarding the perspective, the truthfulness of the image depends on the following distinct variables, related to the principles of geometric optics as applicable to photography [15,16]:

- The focal distance is the distance between the lens’ optic center and the point where the light is concentrated to generate the image inside the camara. Short focal distances increase the relative size of the closest objects compared with the most distant objects, and vice versa.
- The camara–object distance influences the size of the object in the image. Objects further away from the camara are depicted with a more accurate relative size.

In photography, errors of perspective produce the following two phenomena:

- The nearer the object is to the camara, the more distorted it appears [17].
- When a short focal length is used, irrespective of the linear distortions, objects at different distances appear more distanced from each other, and when a long focal length is employed (close to a zoom), objects at distinct differences appear closer to each other [18].

In biomechanics, it is interesting to observe the study object when it is perpendicular to the reference planes of space; that is, the frontal, sagittal, and transverse planes, and mainly the first two. The perpendicularity of the camara to the photographed object is a basic condition when obtaining an orthogonal image. When the perpendicularity cannot be maintained, the data obtained are not reliable, so they must be corrected. Comparative tests have been done between 2D and tridimensional 3D systems, with different results. Among these, the work of Clarke and Murphy (2014) stands out [19]. They compared a 2D video-based movement analysis system (VOHMR) and the VICON 3D Goldstandard^®^. They found sufficient agreement with specific parameters analyzed in the sagittal plane. The same was found by Soda et al. [20]. They designed a bidimensional analysis system for the sagittal plane, double validated by Ugbolue et al. [21], who performed a comparative analysis with a 3D system.

There are thus two problems that need to be resolved:

- To determine the conditions of the video recording for it to be orthogonal, namely, perpendicularity and focal distance, as well as good quality (framing, focus, brightness, contrast, etc.).
- To correct the distortions arising from the lack of perpendicularity in the analysis of the heel (because of the difficulty in obtaining an image in the frontal plane without the other foot interposing itself, and the distinct angulations in the sagittal plane of the rearfoot during the gait) via the regression formula.

We therefore need to dispose of the apparent angle of the rearfoot in the frontal plane, the sagittal plane (which is obtained with a camara perpendicular to the foot) and the transverse plane, which is obtained via a dynamic measurement of the FPA (obtained with a dynamic pedigraph, for example).

Given that the first problem is resolved by following the process of obtaining orthophotographs, this study concerns the second problem.

This study was performed in vitro with a simulator representing the rearfoot in different positions. Ample photographs of these positions were taken to compare the apparent degrees (photograph) with the real ones (placed on the simulator) in such a way that the extent of distortion was known, and the correction procedures could be enacted.

Real angles could be predicted from apparent angles measured from the frames if the planes complementary to those in the photograph were considered (in our case, these would be the sagittal and transverse planes).

## 2. Materials and Methods

This was a transversal experimental study, performed in vitro [22]. No human population was used; rather, we used ranges of the theoretical positions of specific body segments during walking, in this case the positions of the foot. This was simulated in a model in vitro.

**Figure 1:**
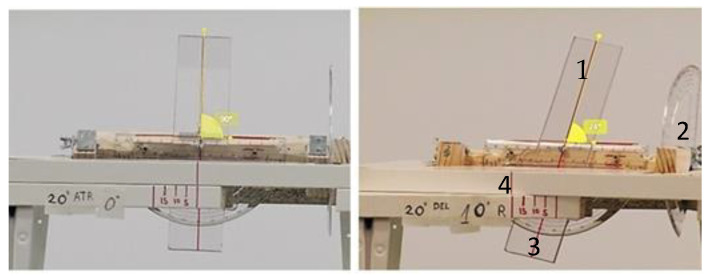
Articulated triplanar goniometric adapted for this study: (1) faithful representation of the hindfoot, (2) sagittal goniometer (3) frontal goniometer, and (4) degree indicator in the sagittal plane.

We designed an “articulated triplanar goniometric simulator” (Figure 1). This device simulates the segment of the rearfoot on which the valgus or varus rearfoot are usually measured during kinematic studies, enabling it to be variously orientated with respect to all of the planes of space. All of the positions of the rearfoot segment were known by the observer, given that the device had a goniometer. For the same position of the rearfoot segment in the frontal plane (quantified in varus or valgus), different photographs were taken (without changing the focus point or the optical parameters) in which the orientation of the segment photographed was modified in other spatial planes (transverse and sagittal), but with maintaining the model in the same degrees of varus or valgus as those that were established at the beginning. The photographs of this model, taken in different postures, were later compared with the real data of the varus or valgus, checking for similarity between the apparent angle (measured in the frame) and the real one. We thus determined the errors the device made in the measurement of the valgus or varus because of the distortion of image caused by the more extreme positions of the model itself in the sagittal or transverse plane. The pointer in the below image represents the rearfoot, and the sagittal goniometer and an indicator of the degrees in the transverse plane are shown as well.

A Casio^®^ EX-F1 (CASIO COMPUTER CO., LTD; Tokyo, Japan) and JVC^®^ GC-PX100BEU (JVC KENWOOD Corporation, Yokohama-shi, Japan) camera was used. The angles in the photographs were measured with Adobe Photoshop^®^ 6.0 software (San José, CA, USA, EE. UU).

The following protocol was used to carry out the measurements. The range of positions used was determined by the following three variables:

- The tilt angle of the back side of the rearfoot in the sagittal plane.
- The gait or FPA in the transverse plane.
- The tilt angle of the calcaneus in the varus or the valgus in the frontal plane.

The data were collected as follows:

- The simulator was placed at a stable point, leveled, and well lit.
- The camara was placed in line with the simulator, at the same height, with its horizontal axis parallel to the ground at a distance of 2.75 m. The zoom was adjusted to 8×. If all of the simulator’s goniometers were adjusted to 0°, the frontal plane goniometer had to remain perpendicular to the camara’s axis.
- The simulator’s sagittal goniometer was adjusted (with a presumed tilt in the back side of the calcaneus in this plane) to 20° behind, and the transverse goniometer (presumed FPA) was adjusted to 0°. A well-lit and focused photograph was taken, and was framed in the frontal plane goniometer, which contained all of the angles of interest in this plane (from 10° varus to 15° valgus).
- The transverse goniometer was moved from 0° to 20°, and a new photograph was taken. Now photographs contained 52 points of extreme data, 26 of 0° transverse and 26 of 20° FPA, all with a sagittal angulation of 20° behind. Table 1 is composed of these data.
- After this, the sagittal plane goniometer was moved from 20° behind to 19° behind, and two photographs of the transverse plane were again taken (one at 0° and the other at 20°).
- This process was repeated for all of the angles of the sagittal plane.
- When all of the photographs had been obtained, the angles of interest were measured (from 15° valgus to 10° varus in the frontal plane) using Photoshop^®^.
- The difference between the values of the model’s real position and those predicted from the photograph indicated the effect of the distortion of the angulations caused by the different orientations of the segment evaluated in the different spatial planes.
- A simple logistic regression model was applied to determine the effect of the distortion of the image and to establish a formula that would correct these distortions whenever the observer knew the tridimensional position of the rearfoot in the image analyzed. IBM SPSS Statistics 22 software (IBM, Armonk, NY, USA, EE. UU.) was used for this.

For the study of interobserver reliability, two more subjects repeated the measurements of 41 images, and the main investigator repeated the measurements of these same 41 images twice more.

The variables of the sagittal plane, the FPA, the angle in the apparent frontal plane, and the angle in the real frontal plane were used for the regression analysis. The aim was to predict the real frontal plane (dependent variable) through the variables of the apparent frontal angle, the FPA, and the sagittal plane. A linear regression analysis was performed with these data.

Model 1: Real frontal plane = α_1_ + α_2_ × apparent frontal plane + α_3_ × sagittal plane + α_4_ × FPA.

In order to maximize the accuracy of the model, based on the reduced significant impact of the sagittal plane and the FPA, a new variable was created that combined both (sagittal plane × FPA). Then, a second model was proposed.

Model 2: Real frontal plane = α_1_ + α_2_ × apparent frontal plane + α_3_ × sagittal plane × FPA.

The study of the reliability and internal consistency employed the interclass correlation coefficient (ICC), Cronbach’s alpha, and the analysis of variance.

## 3. Results

First of all, the statistical consistency of the regression models was determined via an ANalysis Of VAriance (ANOVA) test. This is a statistical technique used to compare the means between three or more groups. Its significant *p*-value indicates that there is at least one pair in which the mean difference is statistically significant [23]. A hypothesis test was carried out based on the following hypotheses: (H0) The obtained regression is not statistically consistent, and (H1) the regression obtained is statistically consistent.

First, regarding Model 1, the ANOVA test presented a *p*-value of less than 0.001, so it can be stated that the regression obtained was statistically consistent, with a linear relationship between variables, at any significance level. An accuracy of 97.1% was obtained in this regression model.

The constant of the equation and the coefficients of the three independent variables (apparent frontal plane, sagittal plane, and FPA) had a significant impact (*p* < 0.001) on the dependent variable (real frontal plane), as is shown in Table 1. The constant and the three variables were incorporated into the model so as to attain the maximum accuracy. The difference of 13 hundredths of a degree (from −0.039 to 0.026) in the interval of the model’s constant was minimal. Via the typical error of the three, an interval with a confidence of 95% could be calculated to estimate both the constant and the three coefficients of the variables, as well as the point estimate. The apparent frontal plane was shown to be the most important predictor variable, with a value of 93.4%.

As was stated in the Methodology section, in order to maximize the accuracy, another model was proposed considering the conjoined effect of the sagittal plane and FPA. This model, Model 2, also showed a significant ANOVA test result (*p*-value < 0.001), meaning that the regression obtained was statistically consistent. An accuracy of 99.9% was obtained in this regression model. The goodness of fit (R squared) was 0.999.

**Figure 2:**
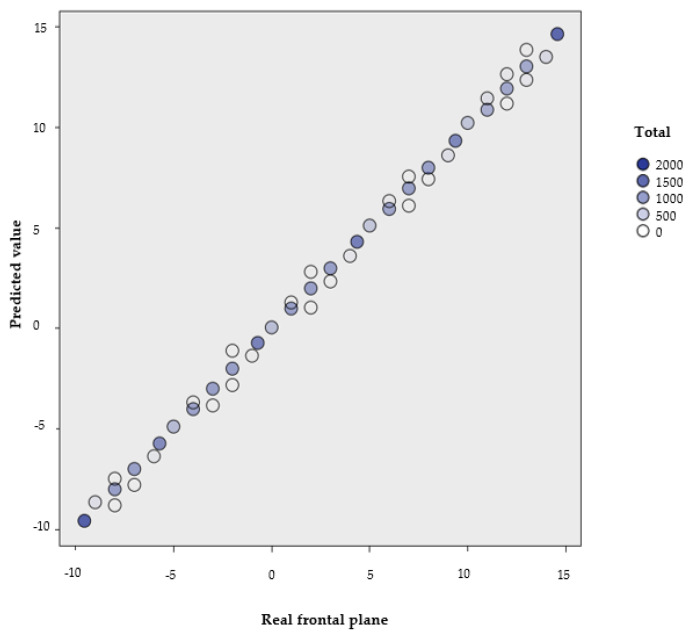
Graphic representation of the predicted values and the observed values in the second and definitive model (Model 2).

In addition, when comparing the predicted values with the real values, an almost perfect diagonal between the two variables was observed, so it can be confirmed that the predictive model is optimally suited for the research (Figure 2).

The constant of the equation and the coefficients of the variables apparent frontal plane and sagittal plane × FPA were consistent in the model (*p* < 0.001), as shown in Table 2. The constant and the two variables were entered into the model to attain the maximum accuracy. The difference of 7 thousandths of a degree (from 0.041 to 0.048) in the interval of the model’s constant was minimal. Via the typical error of the two, a confidence interval of 95% could be calculated to give an estimation of both the constant and the two coefficients of the variables, as well as the point estimate. Apparent frontal plane was shown to be the most important predictor variable, with a value of 91.2%, and the effect of the new variable sagittal plane × FPA was higher than the individual importance of both variables considered jointly, according to Model 1.

In the reliability study, the ICC value of both the individual measurements (ICC: 0.954, CI: 0.954–0:974) and the average measurements (ICC: 0.984, CI: 0.974–0.991) was very good and significant (*p* < 0.001), indicating a high intraobserver reliability. The results corroborated a Cronbach’s alpha of 0.984, indicating the high internal consistency of the measurements taken by the same evaluator. The analysis of variance indicated that there were no significant (0.05) differences between the intraobserver evaluations; in this case, the significance was 0.456.

## 4. Discussion

Since the mid-20th century, human movement analysis has been part of clinical practice [24]. The first studies on gait using quantitative outcome measures were conducted in the 1960s, taking temporal–spatial parameters, the kinematics of limb movement in three planes, and kinetics into account [25]. The images captured through a lens can be submitted to a series of aberrations or distortions, which influence the measurements. To determine the real values of the measurements, the distortion of the image must be considered.

The regression formula was calculated in a prior study, and includes the range of positions of the calcaneus with respect to the ground, from a rear tilt of 20° to a front tilt of −20° forward in the sagittal plane, and from 0° to 20° external tilt in the transverse plane (FPA). Therefore, the proven accuracy (which is very high) is only guaranteed for subjects within these ranges. Nonetheless, the formula is probably also accurate in ranges just outside of the limits of the previous study.

As far as we are aware, no equivalent formula for the rearfoot has been published. The antecedents of this topic are the Kalman filter and the direct linear transformation (DLT) algorithm.

The Kalman filter is an algorithm that resolves the problem of marks not being captured because of occlusion caused by the interposition of other body parts [26].

It uses predictability and probability criteria to estimate the hidden trajectory, considering the possible random alterations (noise) to which the system is subjected. In our case, the algorithm could usefully be applied in bidimensional analyses of the rearfoot, as this is precluded for a significant time by the alignment with the contralateral foot, and it has an open FPA. However, the duration of occlusion is relatively long compared with the gait, and therefore, the degree of uncertainty seems too great for us to trust the estimation of the statistical probabilities. Therefore, while research on this has not yet been done, it should not be ruled out.

Corrector algorithms of the same type as DLT have been used in bidimensional analyses. These apply linear equations that relate the coordinates of a point in the image with its spatial location via the theory of perspective projection [27,28]. For it to be useful to us, at least two coordinated points of view of the study object are needed, with a prior calibration of the field wherein this object was recorded through a tridimensional framework. Subsequent processing of the images with DLT algorithms is also required [29]. Although this is the rough outline, this methodology involves more complex techniques than those that are proposed in our study, and would be beyond the scope of the average clinician or researcher. These are the bases upon which the tridimensional analyses operate.

The objective of the work was to determine the magnitude of the differences between the apparent frontal plane and the real frontal plane, calculating the variability in the real measurements caused by the perspective of the image. The regression formula, which expresses this image distortion in diverse situations of biomechanical interest, shows the variations found between the apparent and the real measurements. Gait cycle is the time from initial contact to initial contact on the same foot, including both the stance phase and swing phase. The stance phase is the period during which the foot is in contact with the support surface within one gait cycle. The swing phase is the period during which the foot is airborne within one gait cycle [30].

Next, these differences in the stance phase’s subphases are explained, namely, loading response, mid-stance, terminal stance, and pre-swing [31,32,33,34].

- Loading response: We are seeking the angle at which the rearfoot meets the ground. The position of the subtalar joint can be deduced from this if the angle between the tibia and the ground is previously known. To this effect, the bisection of the back side of the rearfoot with respect to the ground is measured. According to the results of our study, the magnitude of the potential error is contingent upon the FPA and the deviation in the sagittal plane of the back side of the rearfoot. The FPA is sufficiently well known, but the second factor is not. This depends on three determinants: the inclination of the leg (determined in turn by the stature and the longitude of the stride), the position of the ankle, and the anatomical tilt of the calcaneus.
- Mid-stance: This is the period between the forefoot entering into contact with the ground and the exiting said contact. This will end when the support of the forefoot ends. As in the previous case, there are differences between the apparent frontal plane (photographed) and the real plane. Unlike in the former situation, at this moment, the rearfoot is tilted forwards in the sagittal plane. There is no distortion in the measurements taken in cases of very discrete FPA, or this distortion is at least clinically acceptable. The difference between the apparent measurement and the real measurement is accentuated, especially when the deviation in the sagittal plane exceeds 10°.
- Terminal stance: This is the end of the propulsive period and the beginning of the medium support period. It takes place when all the toes are on the ground. The back side of the forefoot is positioned with a greater tilt forwards in the sagittal plane. The deviation in the sagittal plane depends only on the rearfoot’s morphology. In our studies, we found that no measure without correction is acceptable for an FPA of 10°.
- Pre-swing: This is the end of the period of medium support and the beginning of the propulsive period. At this moment, the subtalar joint must already be supinated, or very close to supination under normal conditions [35]. The regression formula indicates that 5° or less in the FPA is acceptable regarding all of the tilts of the rearfoot in the sagittal plane that do not surpass 20°, provided that we accept that the error is not important from a clinical point of view. It is also confirmed that the greater the conjunction between deviations in the perpendicularity of the FPA and the sagittal plane (in this case 20° and 20°, respectively), the greater the distortion, which, in the maximum expression of all of the ranges studied for all of the frontal planes, is 7°. In this last case, our research is especially necessary.

## 5. Conclusions

Considering the laws of optics, photography, and the peripheral fields investigated, it is possible to take angular measurements of the foot based on the use of two-dimensional images, applicable in biomechanical studies, through which distortions caused by the perspective of the photograph can be corrected.

It is evident that measurements of photos or frames can be a very useful and affordable tool for biomechanical examination if the errors caused by image distortion are considered. The use of this photographic image correction method will be very useful in obtaining more precise data in the context of clinical examination and biomechanics research. It will help us derive a more accurate diagnosis and obtain better treatment results.

Considering the results of this study and already knowing the angles in the transverse and sagittal planes, it is possible to determine the angle of a simulated calcaneus with respect to the ground in the frontal plane applying the regression formula, despite distortions caused by perspective and the lack of perpendicularity.

The measurements taken in this study from different angles on different planes have clear distortion errors in the context of a goniometer simulating a calcaneus. It would be interesting to replicate this procedure on a real foot during walking to validate these measurements. The study of image distortion caused by differences in perspective in a real subject during ambulation can be especially complex because of the difficulty of controlling the triplanar position variables in the segment under study.

## Figures and Tables

**Table 1:** Results of regression Model 1.

Model Term	Coefficient	Standard Error	t	Sig	Confidence Interval 95%		Importance
					Lower	Upper	
Constant	−0.006 (α_0_)	0.017	−0.381	0.703	−0.039	0.026	
Apparent frontal plane	0.986 (α_1_)	0.001	869,072	0.000	0.984	0.988	0.934
Sagittal plane	−0.173 (α_2_)	0.001	−231,338	0.000	−0.174	−0.171	0.066
FPA	0.012 (α_3_)	0.001	0.001	0.000	0.009	0.015	0.000

**Table 2:** Results of regression Model 2.

Model Term	Coefficient	Standard Error	t	Sig	Confidence Interval 95%		Importance
					Lower	Upper	
Constant	0.045 (α_0_)	0.002	26.533	0.000	0.041	0.048	
Apparent frontal plane	1.014 (α_1_)	0.000	46,888.575	0.000	1.014	1.014	0.912
Sagittal plane × FPA	−0.018 (α_2_)	0.000	−1458.431	0.000	−0.018	−0.018	0.088

## Data Availability

The data presented in this study are available on request from the corresponding author.
